# Association between latent tuberculosis and ischemic heart disease: a hospital-based cross-sectional study from Saudi Arabia

**DOI:** 10.11604/pamj.2021.38.362.28110

**Published:** 2021-04-14

**Authors:** Emad Ali Al Khoufi

**Affiliations:** 1 Internal Medicine Department, College of Medicine, King Faisal University, Al-Ahsa, Saudi Arabia

**Keywords:** Ischemic heart, atherosclerosis, latent tuberculosis

## Abstract

**Introduction:**

atherosclerosis could be a sequela of long-term activation of cell-mediated immunity as the case of latent tuberculosis infection. Atherosclerosis is the main pathological event in ischemic heart disease. The present study aimed to assess the prevalence of Latent tuberculosis infection (LTBI) among patients with ischemic heart disease (IHD) and to detect the association between both diseases.

**Methods:**

this cross-sectional study included 98 patients with a history of previously diagnosed ischemic heart disease who did a multi-detector computed tomography coronary angiogram (MDCTCA). Detailed clinical examination and investigations as chest X-ray and sputum examination were done for those with positive QuantiFERON-TB Gold test (QFT) to exclude active tuberculosis (TB). Participants having positive QFT results but with no evidence of active TB were considered as LTBI positive.

**Results:**

the prevalence of LTBI in patients with IHD was 19.3% as only nineteen of the ninety-eight patients were diagnosed with latent tuberculosis infection using the QuantiFERON serum test. Eighty-four percent (84.2%) of patients with LTBI had coronary artery atherosclerosis (CAA) compared to only 55.6% in patients without LTBI with a statistically significant difference. In multivariable analysis, Diabetes Mellitus (DM) (AOR 0.179, 95% C.I.: 0.03-0.967), and LTBI (AOR 1.024, 95% C.I.: 1.002-1.736) were significantly associated with coronary artery atherosclerosis (p=0.0001, and p= 0.003 respectively).

**Conclusion:**

the prevalence of latent tuberculosis infection among patients with ischemic heart diseases is high. Among different factors that are already well known to precipitate ischemic heart disease, latent tuberculosis should be considered.

## Introduction

In developed countries, cardiovascular diseases (CVD) remain the main serious health issue that leads to high mortality rates [1]. Also, coronary artery atherosclerosis (CAA) is a major risk factor for ischemic heart diseases (IHD) [2]. One of the recently discovered theories for the development of atherosclerosis is chronic infection with subsequent development of CVD or stroke [3]. Therefore, many intracellular microorganisms that cause chronic or latent infections as chlamydia, tubercle bacilli, cytomegalovirus (CMV), and human immunodeficiency virus (HIV) were reported to cause atherosclerosis and hence CVD [4]. Several mechanisms were studied to prove the relationship between the presence of infection and the development of CVD. It suggests direct cellular infection with its damage, stimulation of the immune system, or an autoimmunity condition [5]. Moreover, within the same point, various studies proved the relationship between infections within the lungs or urinary system and the development of carotid atherosclerosis [6-8]. Tuberculosis (TB) which is a serious worldwide health problem especially in developing countries, includes a continuous state of inflammation with the production of many cellular inflammatory cells and inflammatory markers as cytokines and chemokines [9,10]. TB and CVD have a high incidence and prevalence worldwide [11,12]. The cascade that starts with inhalation of Mycobacterium tuberculosis and hence stimulation of many inflammatory markers including cytokines and chemokines and ends with atherosclerosis through endothelial, may be the cause of the associated CVD in many patients with TB [13,14]. The second commonly studied mechanism for these links is the induction of autoimmunity. And supposed that chronic infection as TB stimulates autoimmunity with the production of antibodies against mycobacterial heat shock protein-65 in a cross-reaction with human heat shock protein-related to atherogenesis, leading to vascular cytotoxicity and endothelial damage [15]. Latent tuberculosis infection (LTBI) is a TB infection without progression to disease or any clinical manifestation [16,17]. There are no confirmed fixed data about the actual burden of LTBI worldwide. But, nearly 33% of the worldwide population have LTBI [18-20]. It was thought that LTBI is a state of dormancy, but researchers confirmed that this was a wrong concept and in LTBI, mycobacteria remain to replicate and there is a persistent state of immune activity [21]. Moreover, it is proved that in those patients with LTBI, there is a continuous state of monocytes and lymphocytes activation compared with findings in healthy controls [22]. This persistent state of immune activation may be the trigger for atherosclerosis and IHD [23].

A study conducted in Taiwan reported that the risk of acute myocardial infarction and unstable angina increases about 1.4-fold in persons with tuberculosis disease [24]. Similar studies reported that patients with tuberculosis are at risk of ischemic stroke and peripheral artery disease 1.5 and 3.9 times higher respectively than controls [25,26]. Moreover, Huaman *et al*. found that LTBI was independently associated with acute myocardial infarction [27].

The aim of this work was to study the prevalence of LTBI among patients with ischemic heart diseases and to detect if there is any association between both diseases.

## Methods

**Study design and setting:** this cross-sectional study included patients recruited from outpatient cardiovascular disorders clinic and medical records of the patients at King Fahad Hospital (which is a secondary level of care hospital located in Al Ahsa and serving about two million populations) in the period from February 2018 to January 2020.

**Study population:** this study included 98 patients with a history of previously diagnosed ischemic heart diseases (by coronary angiography) who had multi-detector computed tomography coronary angiogram (MDCTCA). Exclusion criteria included incomplete data, patients without MDCTCA, active tuberculosis, previous latent TB treatment, malignancy, chronic renal failure, acute coronary syndrome, and patient treated with immunosuppressants.

**Figure 1:**
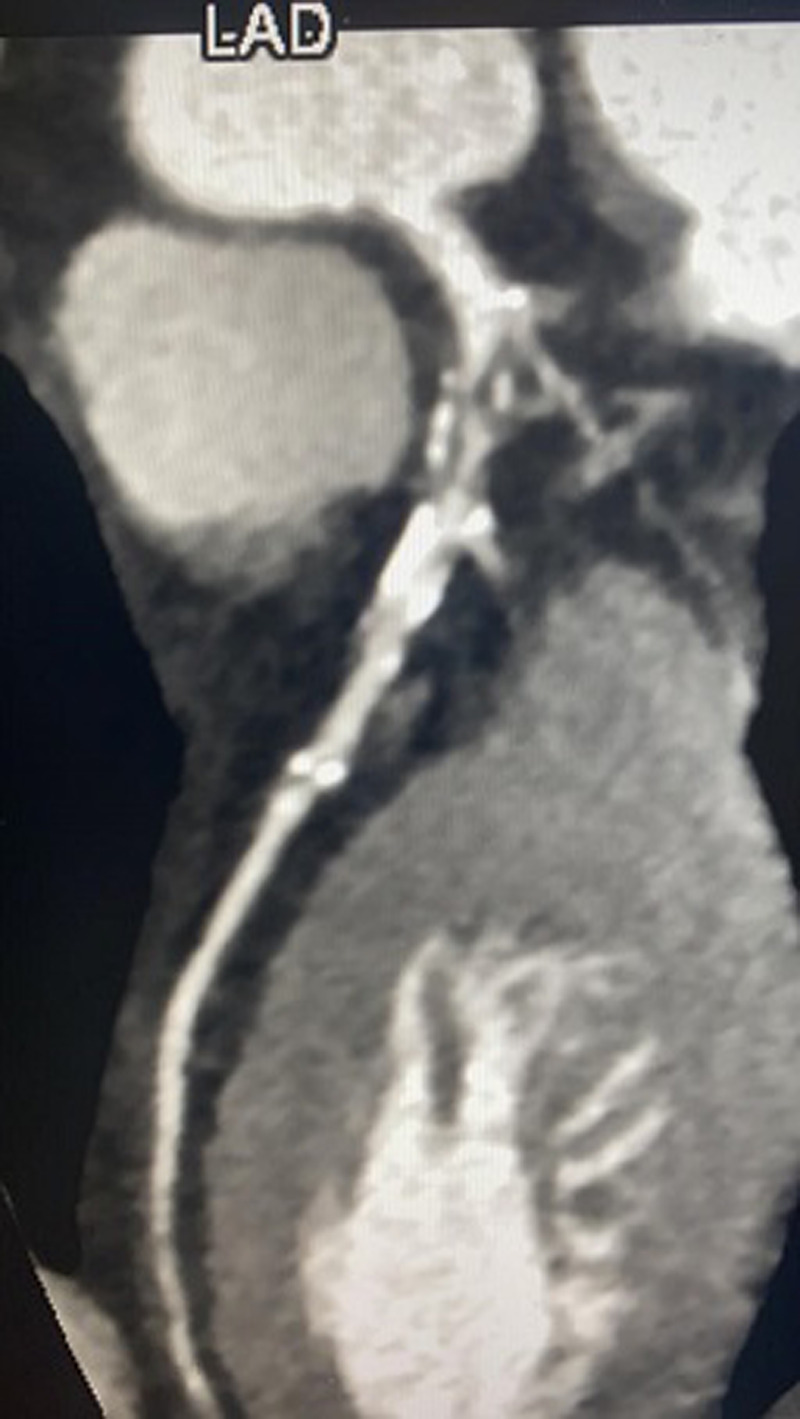
soft and calcific coronary artery disease involving the proximal LAD associated with sever stenosis

**Figure 2:**
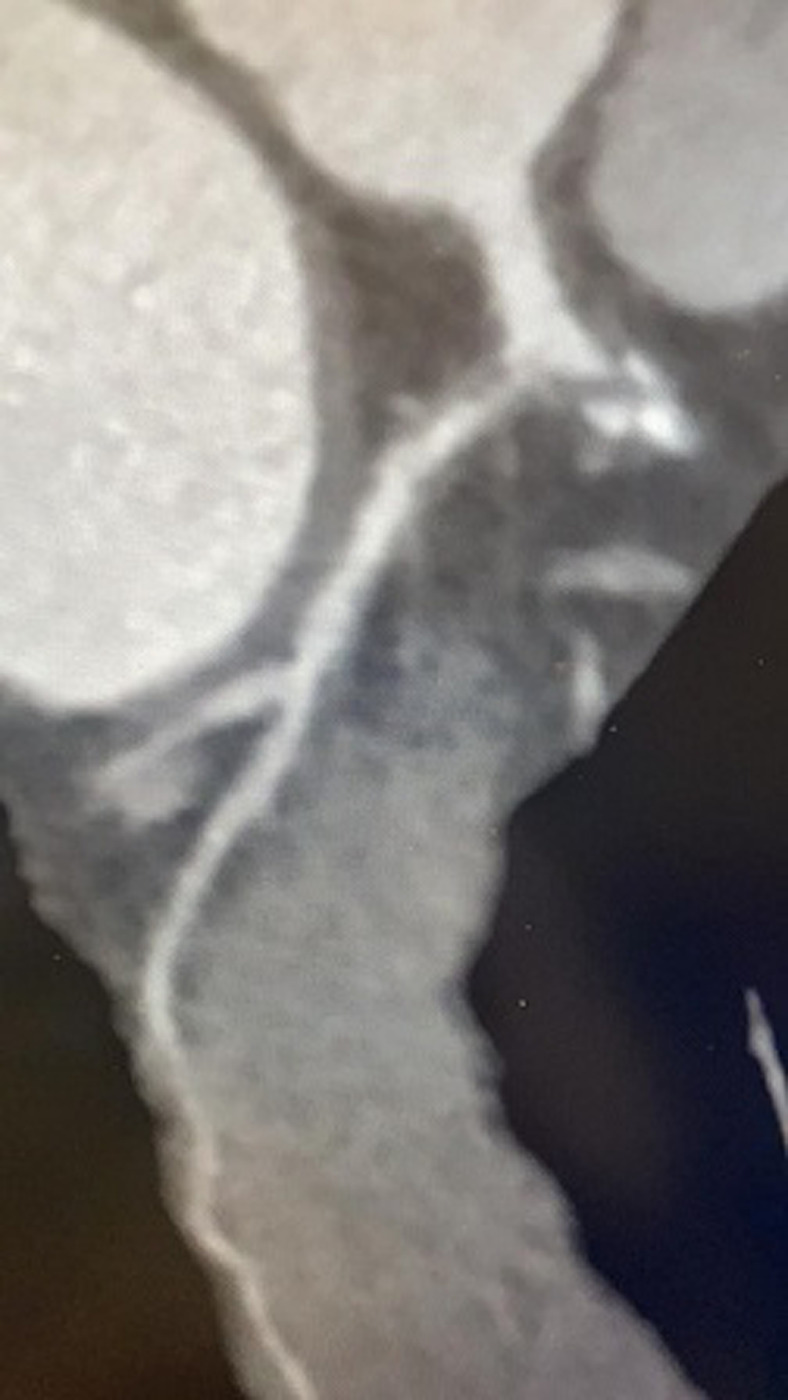
soft plaque involving the proximal LCX associated with sever stenosis

**Data collection:** all patients were subjected to a detailed history taking including age, gender, nationality, smoking index, and history of comorbidities. Clinical examination was done including measurement of height (in m), weight (in kg) and calculation of the body mass index [28]. Laboratory tests included complete blood count (CBC), erythrocyte sedimentation rate (ESR), lipid profile, and blood glucose levels were measured. Dyslipidemia was diagnosed if total cholesterol > 2,70 g/L HDL < 0,44 g/L, LDL > 1,88 g/L or Triglycerides > 1,50 g/L [29]. Obesity was diagnosed as a body mass index (BMI) = 30, whereas a BMI = 25.0 indicates the person is overweight [30]. Moreover, a follow-up echocardiography was done for all patients as well as MDCTCA. For QuantiFERON-TB Gold In-Tube (QFT-GIT) which detects cell-mediated CD8+ cytotoxic T lymphocyte immunologic release of IFN-? with using 3 heparinized blood collection tubes. Three tuberculosis-specific antigens (ESAT-6, CFP-10, and TB7.7) were precoating the first tube. Phytohemagglutinin was precoating the second tube which was used as a positive control (The Mitogen tube) for correct blood handling and incubation, while the third tube was coated with only anticoagulant (negative control tube). Three milliliters of blood were withdrawn prior to tuberculin skin test (TST) administration, incubated at 37°C overnight, and then underwent 10 min centrifugation. Plasma samples were tested by ELISA. According to QFT-GIT (Cellestis Limited, Carnegie, Victoria, Australia), a value = 0.35 IU/mL for {(IFN-ϒ in the TB antigen tube) - (IFN-ϒ in the negative control tube)} was considered a positive result. If the IFN-ϒ level was < 0.35 IU/mL in the TB antigen tube and mitogen control was positive (= 0.5 IU/mL), the test was recorded as negative. If the mitogen control was negative (< 0.5 IU/mL), the test was recorded as an indeterminate result [31]. Patients with positive QuantiFERON-TB Gold test (QFT) were exposed to detailed general and local examination, chest X-ray (CXR), and sputum examination to exclude active TB. Patients with positive QFT with normal examination and investigations were considered positive LTBI. While patients with negative QFT were considered negative LTBI. Those with intermediate results were excluded (12 participants). MDCTCA data were evaluated for the presence of any plaque or significant coronary stenosis (Figure 1, Figure 2). A coronary plaque was identified as a hyperdense structure with or without calcification adjacent to the lumen of any size. And patients were classified regarding the presence or absence of these plaques into patients with and without coronary artery atherosclerosis (CAA) [32].

**Data analysis:** data were analyzed using SPSS version 21 and formulated as the Mean +Standard Deviation (SD). Group differences were analyzed by the Student t test, the Mann-Whitney U Test, and Chi Square for normally distributed, non-normally distributed, and non-continuous variables, respectively, and P values = 0.05 were considered statistically significant. Univariate and multi-variate logistic regression analysis was used to determine the most associated risk factors with coronary artery atherosclerosis which clinically manifested as IHD. In univariate analysis we use the following variables as predictors (age, smoking, obesity, Diabetes Mellitus (DM), Dyslipidemia, LTBI, Hypertension (HTN), gender, and family history) and P values = 0.2 were considered statistically significant.

**Ethical considerations:** this study was approved by the ethical committee of the College of Medicine, King Faisal University, and was done in accordance with the declaration of Helsinki. Written consent was obtained from the patients after receiving proper orientations regarding the study objectives and outcome.

## Results

### General characteristics of the study population

Table 1 shows the general characteristics of the studied group. This study included ninety-eight patients who already diagnosed with ischemic heart diseases through multidetector computed tomography coronary angiogram (MDCT-CA). There were sixty-one males and thirty-seven females with means of age 55±10.05 years old. In addition, there were fifty-one smokers who were either current or former smokers. There were thirty-nine obese patients with BMI = 30. Of the studied patients, 71.42% were hypertensive and 51% had diabetes mellitus. Twelve patients had a family history of ischemic heart diseases. The prevalence of LTBI in patients with IHD was 19.3% as only nineteen of the ninety-eight patients were diagnosed with latent tuberculosis infection using QuantiFERON serum test.

**Table 1:** general characteristics of the studied group (n=98)

Parameter	n (%)
Female	37 (37.7%)
Male	61 (62.3%)
Age (Mean+SD)	55± 10.05
Smoking	51 (52%)
Obesity	39 (39.7%)
HTN	70 (71.42%)
DM	50 (51%)
Dyslipidemia	52 (53.06%)
Family history	12 (12.24%)
LTBI (QFT)	19 (19.3%)

n: number, %: percent, SD: standard deviation, HTN: hypertension, DM: diabetes mellitus, LTBI: latent tuberculosis infection, QFT: QuantiFERON test

### Comparison between patients group according to presence of LTBI and IHD

Table 2 reported the differences between patients with and without LTBI. A higher mean of age was in patients with LTBI (54.91±8.85 years) compared to patients without LTBI (48.66±9.99 years). Regarding gender, there were 11 males and 8 females with LTBI with a non-significant difference. 63.15% of patients with LTBI had Diabetes Mellitus (DM) compared to 48.1% in patients without LTBI. The differences between patients with and without LTBI regarding age and DM were significant (p=0.001 and p=0.05 respectively). There were 57.89% smokers in patients with LTBI and 62.02% smokers in patients without this infection. This table reported non-significant differences between patients with and without LTBI regarding; gender, smoking, HTN, dyslipidemia and obesity (p>0.05). Also, Table 2 shows that, 84.2% of patients with LTBI had CAA compared to only 55.6% in patients without LTBI with statistically significant difference (p=0.05).

**Table 2:** comparison between patients with and without latent TB

Parameter		LTBI (19)	Non-LTBI (79)	P value
**Age (years) Mean+SD**		54.91+8.85	48.66+9.99	0.009
**Gender**	Male	11	50	0.14
	Female	8	29	
**Smoking (current and former) %**		11 (57.89%)	49 (62.02%)	0.91
**DM (%)**		63.15%	48.1%	0.04
**Obesity (%)**		58.34%	62.11%	0.29
**HTN (%)**		68.42%	69.67%	0.46
**Dyslipidemia (%)**		43.27%	49.87%	0.16
**Atherosclerosis (%)**		84.2%	55.6%	0.02

n: number, %: percent. SD: standard deviation, HTN: hypertension, DM: diabetes mellitus, LTBI: latent tuberculosis infectitfon

Table 3 shows that coronary atherosclerosis was detected in 63 patients of this study with MDCT-CA. Also, this table reported a higher mean of age in patients with IHD compared to patients without atherosclerosis; 53.44 and 46.62 years respectively and this difference was statistically significant (p=0.001). Regarding gender, there were 41 males and 19 females in patient with IHD compared to 20 males and 18 females in patients without IHD. However, without any significant differences (p>0.05). There were 63.49% smokers in patients with IHD and 31.4% smokers in patients without IHD. 60.3% of patients with IHD had DM compared to 34.2% in patients without IHD. Moreover, 80.9% of patients with IHD had HTN compared to 54.2% in patients without IHD. Also, 68.2% of patients with IHD had dyslipidemia compared to only 25.7% of patients without IHD. These differences in HTN, DM, dyslipidemia and smoking between patients with and without IHD were statistically significant differences (p=0.05). While there were non-significant differences between both groups regarding prevalence of obesity and family history of ischemic heart diseases (p>0.05). 25.39% of patients with IHD had LTBI compared to only 8.5% in patients without IHD, and this difference shows a statistically significant difference (p=0.05).

**Table 3:** comparisons between patients with and without coronary artery atherosclerosis (CAA)

Parameter		CAA (n=63)	Non-CAA (n=35)	P value
**Age (years) Mean+SD**		53.44 ± 5.98	46.62 ± 7.84	0.001
**Gender**	**Male**	41	20	0.81
	**Female**	19	18	
**Smoking (current& former)**		63.49%	31.4%	0.002
**DM %**		60.3%	34.2%	0.043
**Obesity %**		47.6%	25.7%	0.06
**HTN %**		80.9%	54.2%	0.01
**Dyslipidemia%**		68.2%	25.7%	0.04
**Family history of CAD %**		12%	11 %	0.23
**LTBI %**		25.39%	8.5%	0.03

n: number, %: percent, SD: standard deviation, HTN: hypertension, DM: diabetes mellitus, LTBI: latent tuberculosis infection

### Univariate and multi-variate analysis of risk factors for IHD

In univariate analysis, we use the following variables as risk factors for IHD (age, smoking, obesity, DM, Dyslipidemia, LTBI, HTN, gender, and family history.) Only DM (AOR 0.179, 95% C.I.: 0.03-0.967) and LTBI (AOR 1.024, 95% C.I.: 1.002-1.736) were significant associated with Ischemic heart disease (p<0.05). So, we include DM and LTBI especially in multivariate analysis as shown in Table 4.

**Table 4:** univariate and multivariate logistic regression analysis of all factors associated with coronary artery atherosclerosis

	Univariate			Multivariate		
	AOR	(95% CI)	P- Value	AOR	(95% CI)	P- Value
**Age (years)**	1.178	(0.772, 1.963)	0.625			
**Smoking**	1.128	(0.196, 1.327)	0.238			
**Obesity**	1.034	(0.009, 2.307)	0.409			
**DM**	1.093	(1.037, 1.896)	0.001	0.179	(0.03, 0.967)	0.0001
**Dyslipidemia**	1.712	(0.731, 3.947)	0.322			
**LTBI**	1.891	(1.010, 1.936)	0.0001	1.024	(1.002, 1.736)	0.003
**HTN**	0.439	(0.258, 1.885)	0.360			
**Gender**	0.658	(0.133, 1.208)	0.418			
**Family history**	1.002	(0.662, 1.603)	0.528			

AOR: adjusted Odds Ratio, CI: confidence interval, HTN: hypertension, DM: diabetes mellitus, LTBI: latent tuberculosis infection

## Discussion

During the period of our study, ninety-eight patients diagnosed with ischemic heart diseases by multidetector computed tomography coronary angiogram were investigated for latent TB infection by using QuantiFERON-TB Gold In-Tube. Out of 19 patients with LTBI, 84.2% had CAA. Mean age of patients with CAA was 53.44±5.98 versus 46.62±7.84 in patients with non-CAA. (P value<0.001) The impact of age as a common risk factor for ischemic heart diseases was supported with Dhingra and Vasan [33] who stated that there are a lot of cardiovascular disorders events that are acquired with the increase of age during any individual whole life. They also reported that increased age was an independent risk factor associated with these disorders. Moreover, age is a fixed risk factor that is present in all risk stratifications for cardiovascular diseases assessment and is associated with increased risk of cardiovascular disorders events [33]. Sixty-two (62.3)% of patients enrolled in the study were male and this is in agreement with different studies [34-36] reported that before the age of sixty, males have 3-4 folds increase in the prevalence and incidence of different coronary artery syndromes including angina and myocardial infarction. In agree with the previous researches [34-36] male is the predominance gender in patients with IHD. Regarding other comorbidities hypertensive was the most prevalent (71.42%) followed by dyslipidemia (53%), smoking (52%) and diabetes mellitus (51%). This is in aligned with well-known risk factors of IHD [37] Among patients diagnosed with latent tuberculosis we reported a higher significant of age and prevalence of DM which this is in agreement with that of Cousins [38] and Martinez *et al*. [39] who documented the association between poor blood glucose level control and LTBI. In addition, they stated that an elevated glucose level is associated with increased LTBI. In the same context, Jackson *et al*. [40] and Barron *et al*. [41] reported significant association between increasing age and LTBI. In our work, coronary atherosclerosis (CAA) was reported 63 cases. Those patients had a higher mean of age and higher prevalence of HTN, DM, dyslipidemia and smoking compared with patients without CAA. These findings are in agreement with various studies which stated cigarette smoking, dyslipidemia and hypertension are associated with increased coronary atherosclerosis that was detected with computed tomography angiogram or invasive coronary angiogram and this increase in the process of CAA leads to increased CVD [42-44].

Infection may contribute to atherogenesis and acute cardiovascular events through different mechanisms [45]. Similar to what has been described in other chronic infections, such as HIV infection [46], persistent immune activation related to intermittent low-level microbial replication is a possible driver of the association between LTBI and AMI [47]. Supporting this hypothesis, studies indicate that there is ongoing M. tuberculosis replication and metabolic activity during LTBI [21]. Unlike the former view of LTBI as a state of mycobacterial dormancy, LTBI is now recognized as a continuous continuum of host-pathogen interactions in which replicating and metabolically quiet mycobacterial populations coexist and are restricted by variable host immune responses within each granuloma [48]. Our research reported that the prevalence of LTBI in patients with IHD was 19.3% and 84.2% of patients with LTBI had coronary artery atherosclerosis. Moreover, 25.39% of patients with coronary artery atherosclerosis had LTBI compared to only 8.5% in patients without CAA. This shows a statistically significant difference. Using a multi-variate logistic regression analysis shows that among the significantly correlated factors with CAA, LTBI is among the factors that are associated with CAA. Tuberculosis (TB) which is a serious worldwide health problem especially in developing countries, includes a continues state of inflammation with the production of many cellular inflammatory cells and inflammatory markers as cytokines and chemokines [9,10]. Atherogenesis has been linked to several infectious agents (including viral, bacterial, and parasitic pathogens) through both direct and indirect mechanisms [49]. Mycobacterial direct invasion of the wall of blood vessels leading to an autoimmune reaction, and subsequently thrombosis, is the direct mechanism. Mycobacterium´s cell wall indirectly induces a pro-inflammatory state, which can eventually lead to atherogenesis [48]. Moreover, it is proved that in those patients with LTBI, there are a continues state of monocytes and lymphocytes activation. Compared with findings in healthy controls, LTBI has been associated with enhanced lymphocyte and monocyte immune activation [22]. This persistent state of immune activation may be the trigger for atherosclerosis and IHD [23]. The association between LTBI and CAA reported in this study is aligned with the case-control study that stated that recent AMI was independently associated with an approximately 2-fold increased odds of LTBI, after adjustment for established CVD risk factors and other potential confounders. As expected, known CVD risk factors such as male sex and tobacco use were also associated with AMI [49]. Our study also emphasizes what is stated recently that increased risk of IHD is independently correlated with LTBI and LTBI is considered a non-traditional risk correlation to coronary atherosclerosis [50,51].

The current study has some limitations like, the prevalence of LTBI in studied patients may be affected by the exclusion criteria especially those who did not have MDCTCA and have incomplete data. Also, the diagnosis of coronary artery atherosclerosis is based on the reports of MDCTCA which lead to the missing of some positive cases for CAA.

## Conclusion

The prevalence of latent tuberculosis infection among patients with ischemic heart diseases is high. Among different factors that well known to precipitate to coronary atherosclerosis, latent tuberculosis should be considered and suspected especially if associated with DM and old age. Further studies should be done to evaluate the relationship between LTBI and systemic atherosclerosis especially cerebral atherosclerosis but with using case-control studies.

### What is known about this topic

- Infections are risk factors for cardiovascular diseases;
- TB and CVD have a high incidence and prevalence worldwide.

### What this study adds

- The prevalence of latent tuberculosis infection among patients with ischemic heart diseases is high;
- Latent tuberculosis should be considered among different factors that are already well known to precipitate coronary atherosclerosis
